# Giant controllable gigahertz to terahertz nonlinearities in superlattices

**DOI:** 10.1038/s41598-020-72746-5

**Published:** 2020-09-29

**Authors:** M. F. Pereira, V. Anfertev, Y. Shevchenko, V. Vaks

**Affiliations:** 1 Department of Physics, Khalifa University of Science and Technology, 127788 Abu Dhabi, UAE; 2 Institute of Physics, Czech Academy of Sciences, 18221 Prague, Czech Republic; 3 Institute for Physics of Microstructures, Federal Research Center Institute of Applied Physics of RAS, GSP-105, Nizhny Novgorod, Russia 603950; 4 Lobachevsky State University, 23, Gagarin Av., Nizhny Novgorod, Russia 603950

**Keywords:** Electronic properties and materials, Semiconductors, Surfaces, interfaces and thin films

## Abstract

Optical nonlinearities are of perpetual importance, notably connected with emerging new materials. However, they are difficult to exploit in the gigahertz–terahertz (GHz–THz) range at room temperature and using low excitation power. Here, we present a clear-cut theoretical and experimental demonstration of real time, low power, room temperature control of GHz–THz nonlinearities. The nonlinear susceptibility concept, successful in most materials, cannot be used here and we show in contrast, a complex interplay between applied powers, voltages and asymmetric current flow, delivering giant control and enhancement of the nonlinearities. Semiconductor superlattices are used as nonlinear sources and as mixers for heterodyne detection, unlocking their dual potential as compact, room temperature, controllable sources and detectors. The low input powers and voltages applied are within the range of compact devices, enabling the practical extension of nonlinear optics concepts to the GHz–THz range, under controlled conditions and following a predictive design tool.

## Introduction

Nonlinear physics is playing a major role in bridging what was once called the “terahertz gap” and its multitude of applications^1,3^. As a matter of fact, enhancements of nonlinearities, such as High Harmonic Generation (HHG) are of perpetual interest in nonlinear optics, notably when new materials become available, e.g. graphene, quantum and hybrid materials; even more so if THz operation is possible^1–8^. Quantum confined systems, such as semiconductor superlattices (SSLs) further enhance the intrinsic nonlinearities of materials^9^. SSLs are epitaxially grown by alternating layers of two semiconductor materials with similar lattice constants, creating a spatially periodic potential for electrons^10,11^. This leads to highly nonparabolic energy dispersions, which inspired the early work of Esaki and Tsu^12^ to predict the possibility of frequency multiplication. The demonstration of Stark ladders and coherent Bloch oscillations^13^ paved the way for SSL multipliers (SSLMs) to provide radiation up to the 54th harmonic at 8.1 THz as well as for their use in heterodyne detection with applications to high-resolution spectroscopy^14,15^. Compact high performance superlattice electron devices (SLEDs) have recently reached a record room temperature 4.2 mW power output in the fundamental mode at 145 GHz^16^ and synchronization between SSLs leads to a dramatic increase in output power^2^. In both cases there are propagating charge domains that produce powerful high-frequency GHz radiation and which are triggered by negative differential conductivity (NDC). If we consider integrating SLEDs on chip with SSLMs a new generation of compact sources can lead to low cost, efficient hand held devices for many other applications, including various types of GHz-THz spectroscopy/microscopy. Reference^17^ summarizes the main competing technology for the THz-Mid Infrared range, i.e. Quantum Cascade Lasers where the optical response is due to transitions between well-defined sub-bands.

References^18,19^ summarize the realistic HHG efficiencies that can be achieved by coupling unbiased SSLMs with the most relevant commercially available compact GHz input sources. These type of devices exploit the radiation at odd harmonics of the input oscillating field, originating from the phase-modulated Bloch oscillations and under the assumption of a uniform electric-field distribution across the SSL sample. In the present paper we show that higher outputs can be obtained for the even harmonics with applied biases.

Recently it has been shown that SSLs with asymmetric current flow can deliver not only the expected odd harmonics, but also even harmonics, without a symmetry-breaking static bias^6,7^. The symmetry-breaking effects were attributed to different interface roughness scattering rates^6^. The next and far more complex step is thus to address the challenge of how asymmetric flow, voltage and power can be combined to control and enhance the even harmonics. Previous attempts to control nonlinearities in a SSL with voltage required a high power (input power ${P}_{0}\approx 5$ W) free electron laser GHz input, were restricted to the second and third harmonics and could not explain asymmetries in the power output^8^. Also recent studies of giant enhancement of harmonic generation were still well described by a susceptibility concept related to subband transitions in quantum cascade laser structures. The maxima/minima of output were resolved spectrally and would need a change in sample for a variation^4^. Furthermore, the experiments were realized at 10 K, while ours is a direct control room, temperature demonstration. Note also that the very interesting studies of control of THz high-harmonic generation by dynamical Bloch oscillations require super intense THz pulses and control was actually at pulse generation level requiring large energy fs-source^20^. Nonlinear crystals have delivered pulsed, tunable GHz to THz sources, but also relied upon large pulsed sources^21–24^.

In contrast, here we deliver for the first time a clear cut theoretical and experimental demonstration of the strong and unexpected interplay between asymmetric flow, applied static voltages and oscillating input power that occur when Bloch oscillations in a SSL are modulated by a GHz field with output in the GHz-THz range. This leads to giant changes in the nonlinear output, detected as HHG up to 6th harmonic, instead of a monotonic increase of output with increasing power, as expected from the usual nonlinear susceptibility concept. This effect is demonstrated experimentally by means of an innovative scheme with the same type of SSLs operating both as nonlinear source and heterodyne mixer, delivering a wide frequency span in real time, low powers and room temperature operation altogether.

We have estimated the efficiency of the SSLM multiplier under voltage by means of a calibration method explained in the “Methods” section. For the 4th Harmonic and an intrinsic input power of 291 μW an output of 2.3 μW is achieved, corresponding to 8% intrinsic efficiency. For the 4th Harmonic and an intrinsic input power of 614 μW an output of 2.4 μW is achieved, i.e. 4% intrinsic efficiency. This is about 3 orders of magnitude better than the intrinsic efficiency of previous measurements without the voltage control^6,7^ where an intrinsic input power of 47 μW led to 4.75 nW and 3.83 nW of output respectively for the 4th and 6th harmonics, corresponding to intrinsic efficiencies of 0.01% and 0.008%.

## Results and discussion

### Nonlinearities and high harmonic generation in semiconductor superlattices

**Figure 1:**
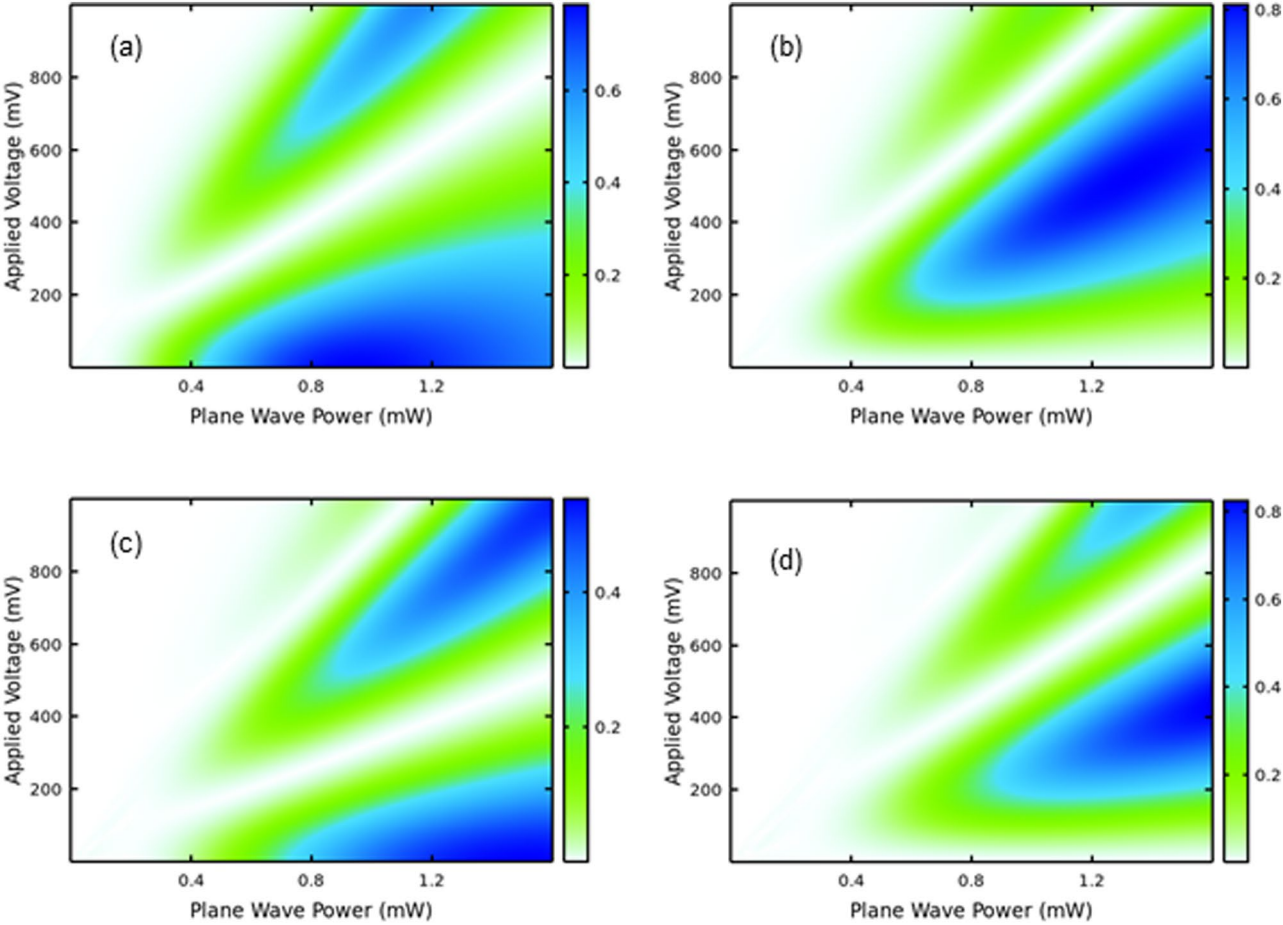
Voltage and power control of Harmonic Generation in Superlattices. The surface plots show the calculated normalized harmonic output power as a function of GHz input power and applied static bias. In all plots the harmonic power is generated by a GaAs–AlAs superlattice with period d = 6.23 nm excited by an oscillating field with input frequency ν = 120 GHz. From (**a**–**d**) the harmonics are, respectively the 3rd, 4th, 5th and 6th. In each panel the color bar is normalized to the maximum power output within the (0, 1000) mV range.

If the (nonlinear) current–voltage flow is perfectly antisymmetric, as expected for an ideal SSL^12,13^, even harmonics of an input frequency can only develop with an applied bias ${E}_{dc}\ne 0.$ If the flow is not perfectly antisymmetric, they appear even at zero bias, as confirmed by the steady state experiments of Refs.^7,8^. In conventional nonlinear optics, the power emitted by mechanisms described by susceptibilities increase with the input field interacting with nonlinear media, since the generated harmonic field amplitude has a structure ${E}^{(n)}={\chi }^{(n)}{E}_{input}^{n}$. Figure 1 shows that, in contrast to the conventional ${\chi }^{(n)}$ approach, increasing the pump does not lead to maximum output and for high voltages, the maxima develop into “petals”, with well-defined maxima and minima.

**Figure 2:**
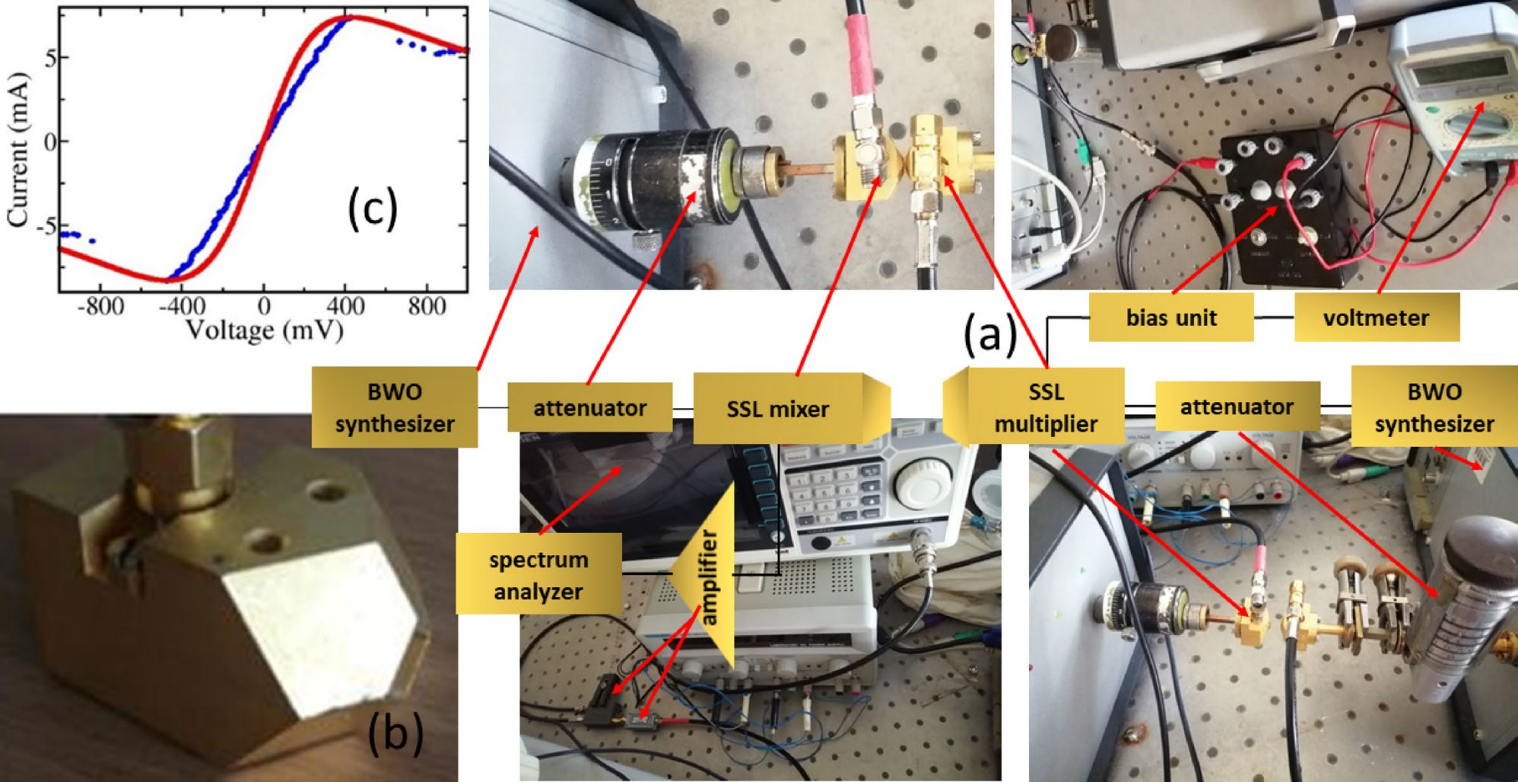
(**a**) Diagram of the experimental scheme, showing pictures of the main units used. (**b**) Close up of the waveguide housing both multiplier and mixer superlattices. (**c**) Current voltage used to extract input parameters for our modelling: the (blue) symbols are experimental data and the (red) solid curve is calculated.

The experimental scheme used in our study is summarized in Fig. 2.

**Figure 3:**
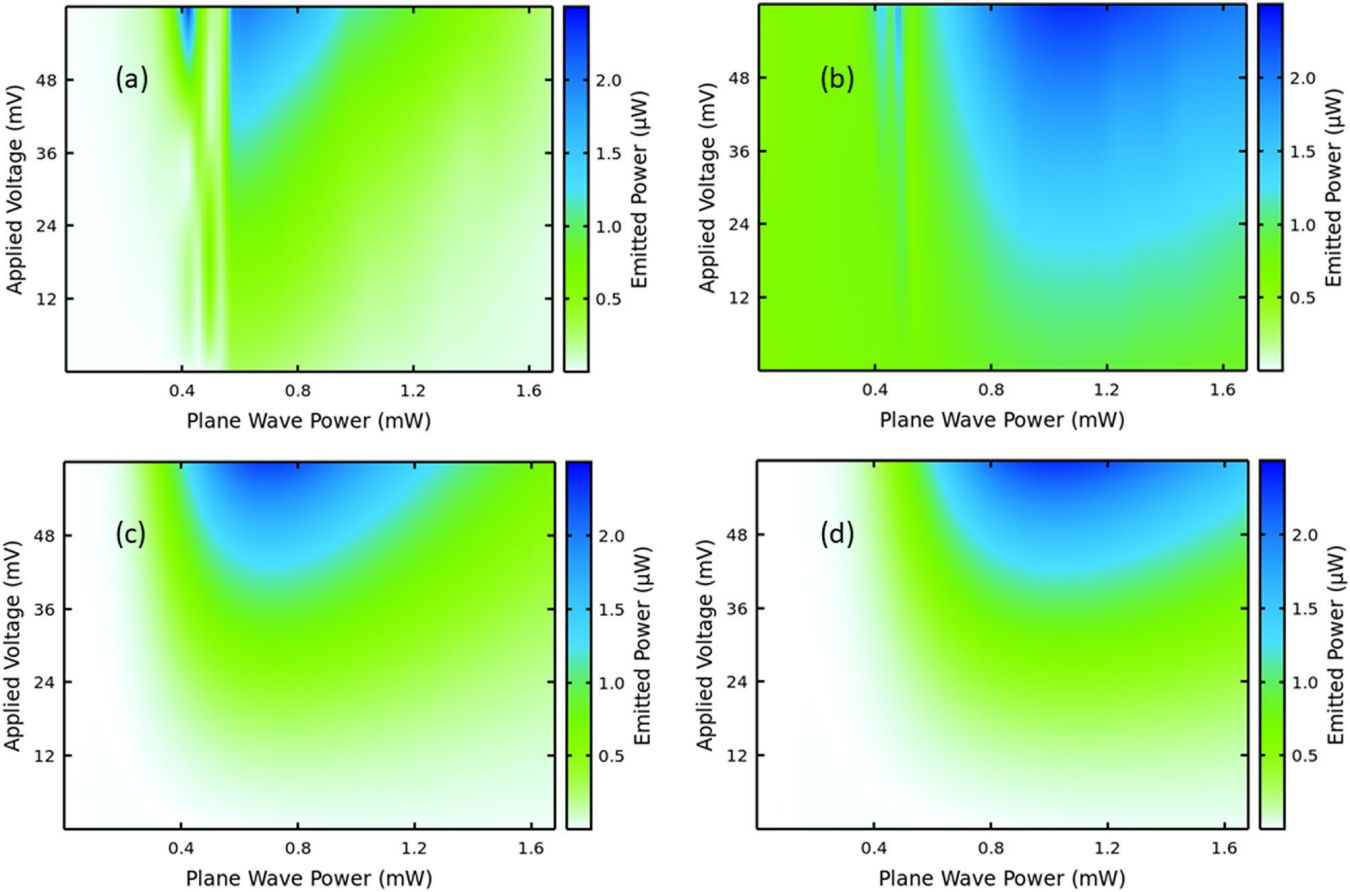
Experimental demonstration of voltage and power control of nonlinearities. Measured (**a**, **b**) and calculated (**c**, **d**) [fourth (**a**, **c**) and sixth (**b**, **d**) harmonics] emitted powers for an input field oscillating at $\nu \hspace{0.17em}=\hspace{0.17em}$120 GHz. The input and output power detected by our heterodyne technique have been calibrated by simulated annealing as explained in the “Methods” section.

Our experiments in Fig. 3a,b confirm the theoretical predictions of Fig. 3c,d that maxima develop in well-defined regions in the applied voltage-input power plane.

The main equations from Refs.^7,8^ used to predict and understand the data are given below.

The nonlinear response, detected as harmonic generation to a GHz input, results from the strongly nonlinear current–voltage (I–V) characteristic of the SSL. The system is excited by electric fields, which consist of static and oscillating parts, $E\left( t \right) = { }E_{dc} + E_{ac} \cos \left( {2\pi \nu t} \right)$. For this combined input field, which is parallel to the growth direction of the SSL with period ${ }d$, the general current response can be written as^7,8^.

1 $$\begin{aligned} & I\left( t \right) = I_{dc} + \mathop \sum \limits_{l = - \infty }^{\infty } I_{l}^{c} \cos \left( {2\pi l\nu t} \right) + I_{l}^{s} \sin \left( {2\pi l\nu t} \right), \\ & I_{dc} = \mathop \sum \limits_{p = - \infty }^{\infty } J_{p}^{2} \left( \alpha \right)Y\left( U \right), \\ & I_{l}^{c} = \mathop \sum \limits_{p = - \infty }^{\infty } J_{p} \left( \alpha \right)\left[ {J_{p + l} \left( a \right) + J_{p - l} \left( a \right)} \right]Y\left( U \right), \\ & I_{l}^{s} = \mathop \sum \limits_{p = - \infty }^{\infty } J_{p} \left( \alpha \right)\left[ {J_{p + l} \left( a \right) - J_{p - l} \left( a \right)} \right]K\left( U \right), \\ \end{aligned}$$

where d is the SSL period, ${ }J_{p}$ is the Bessel function of the first kind and order ${ }p$. $U = eE_{dc} d + p{\text{h}}\upnu$ is the resulting effective potential difference which electrons experience instead of the bare potential due to the dc bias. The electron charge is denoted by ${ }e$. If the distribution of electrons is approximately homogeneous, the local transport properties are governed by the global voltage-current characteristic of the device. The parameter $\alpha = eE_{ac} d/{\text{h}}\upnu$ (h is Plank’s constant), which appears automatically as a consequence of our model controls the nonlinear response of the system and its strong deviation from typical N-order susceptibilities. The functions $Y$ and ${ }K$ which hold for the miniband transport within the relaxation time $\tau$ approximation, are given by

2 $$Y\left( U \right) = j_{0} \frac{2U/\Gamma }{{1 + \left( {U/\Gamma } \right)^{2} }},K\left( U \right) = { }\frac{{2j_{0} }}{{1 + \left( {U/\Gamma } \right)^{2} }}.$$

Here, $\Gamma = \hbar /\tau { }$ is the scattering induced broadening and ${ }j_{0}$ is the peak current corresponding to ${ }U = U_{c} { }\left[ {U_{c} \equiv \Gamma } \right]$. Usually we calculate both quantities with a NEGF approach and insert them in the Equations above. Asymmetric flow is taken into account using the Ansatz introduced in Ref.^7^.

3 $$j_{0} = \left\{ {\begin{array}{*{20}c} {j_{0}^{ - } , U < 0} \\ {j_{0}^{ + } , U \ge 0} \\ \end{array} } \right.,\quad \Gamma = \left\{ {\begin{array}{*{20}c} {\Gamma^{ - } , U < 0} \\ {\Gamma^{ + } , U \ge 0.} \\ \end{array} } \right.$$

The main parameters extracted from the experiments, see Fig. 2c, used in this ansatz solution are: $j_{0}^{ + } ,{ }j_{0}^{ - }$ = 7.4, 8.3 mA and $\Gamma^{ + } ,{ }\Gamma^{ - }$ = 23.88, 26.06 meV. These are energy drop per period and there are 18 periods, each period has a length $d =$6.23 nm. The average power emitted by lth Harmonic is calculated from the Poynting vector

4 $$P_{l} \left( \nu \right) = {\mathcal{T}}\left( \nu \right){ }\left\{ { < I\left( t \right){\cos}\left( {2\pi l\nu t} \right) >^{2} + < I\left( t \right){\sin}\left( {2\pi l\nu t} \right) >^{2} } \right\},$$

where ${ }I\left( t \right)$ is the current in Eq. (1), induced in the SSL by the total field ${ }E\left( t \right)$ and the averaging $< \cdot\cdot\cdot >_{t}$ is performed over the period ${ }T = 1/\nu$. ${\mathcal{T}}\left( \nu \right)$ is the waveguide transmission. In all plots in each panel the normalized power shown is ${\mathcal{P}}_{l} \left( \nu \right) = P_{l} \left( \nu \right)/P_{l}^{max} { }\left( \nu \right)$.

The connection between the driving parameter $\alpha$ and the plane wave power shown in the figures below is $P_{in} \left( {\text{W}} \right) = 29.6 \times 10^{ - 13} \nu^{2} \alpha^{2} .{ }$ The input frequency is $\nu =$ 120 GHz.

**Figure 4:**
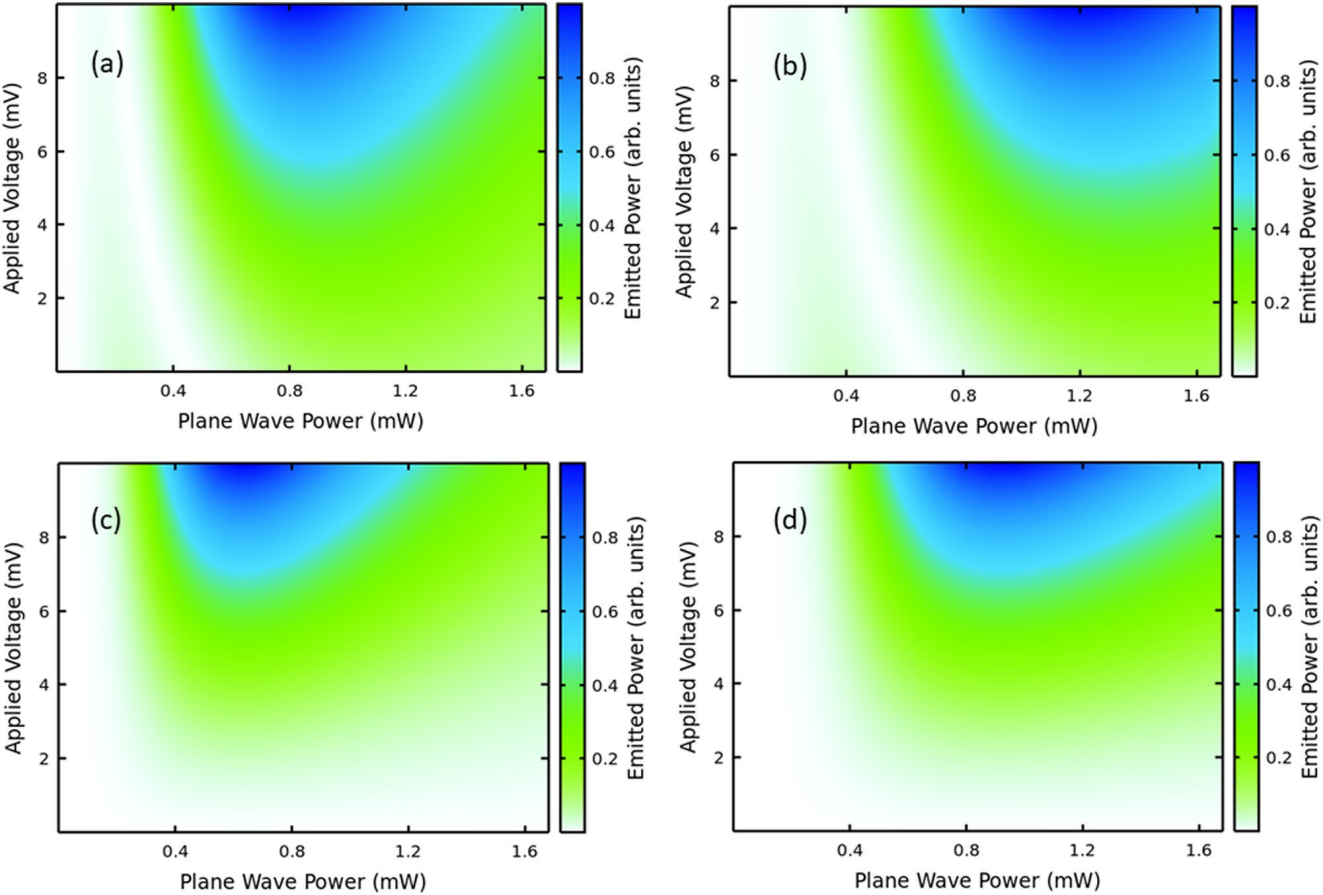
Asymmetric versus Symmetric current flow. Calculated harmonic powers under asymmetric (**a**, **b**) versus symmetric (**c**, **d**) current flow for the fourth (**a**, **c**) and sixth (**b**, **d**) harmonics with an input field oscillating at $\nu$=120 GHz. The input has been calibrated by simulated annealing as explained in the “Methods” section. In each panel the color bar is normalized to the maximum power output within the voltage range (0, 10) mV. The goal here is to show the clear that there is no emission around zero voltage for symmetric current flow in contrast with the asymmetric flow case.

The experiments have been performed with GHz input leading to THz output, demonstrating full control and enhancement of the nonlinearities in terms of applied voltage and power, in a regime that can be studied with input fields from compact devices^19,20^. A clear-cut proof of the role of asymmetric flow in our own data is highlighted in Fig. 4, where we zoom on the low voltage range of the graphs. Each panel is normalized to the maximum emission value in the range displayed. The symmetric curves are calculated with ${\Gamma }^{ + } = {\Gamma }^{ - } =$ 23.88 meV and $j_{0}^{ + } = j_{0}^{ - } =$ 7.4 mA.

### Origin of the nonlinearity and deviations from the ideal model

The experiments in Fig. 3 clearly demonstrate that the nonlinearities can be controlled and enhanced as predicted by the theory, but note that the structural defects, imperfect interfaces^7,8,19,20^ and possible charge field domains make the experimental I–V different to the ideal I–V used in the theory, see Fig. 2c, so it is normal that the nonlinear response would have some quantitative deviations from the ideal solution. First of all, the imperfect coupling prevents the full incident GHz power in the waveguide to reach the superlattice. We have used a calibration described in the “Methods” section to determine how much power reached the superlattice.

Furthermore, a limitation in the system that reflects on deviations from the theoretical predictions is that for the same input power, high harmonics in SSLMs deliver smaller output power than, e.g. the second or third harmonics, making them harder to detect with conventional THz detectors. For a concrete example, note that the power output due to harmonic generation in a SSLM operating without bias has been recently measured^6,7^. The values detected for the 3rd, 4th, 5th, 6th and 7th harmonics for 141 GHz input were given respectively by 3.30 × 10^−7^, 4.75 × 10^−9^, 5.28 × 10^−9^, 3.83 × 10^−9^ W. We see that the higher harmonics suffer a significant decrease in emitted power compared to the third harmonic. Theoretically, at large amplitudes of the oscillating field ($eE_{ac} d \gg h\nu { })$, the Bloch oscillations can contain harmonics of a higher order as a result of the large number of Bragg reflections during the field period. For instance, the lth harmonic which is proportional to ${ }J_{l} \left( {eE_{ac} d/h\nu } \right)$, is largest in the field with amplitude $E_{ac} \sim \left( {l + 1} \right)h\nu /ed$ and therefore we naturally anticipate a lower power at a higher harmonic, for a fixed value of $E_{ac}$.

However, due to the negative differential resistance of the SSL at optimal local oscillator power, our method is sensitive up to the 6th harmonic at room temperature. Thus, we manage to overcome the low power, the high conversion losses of multiplier and mixer at high harmonics and finally the extra noise by the SSL frequency mixer in our heterodyne detection scheme. As a matter of fact, at this point, we should briefly summarize different mechanisms that are known to contribute to nonlinearities in SSLs and can help explain further discrepancies between our model and the experiments. Frequency multiplication happens when the Bloch-oscillating electron wave packet is driven by an input oscillating field^3,9^. This output due to the frequency modulation of the Bloch oscillations takes place in the negative differential conductivity (NDC) region of the current–voltage curve^3^. Note that also that if a SSL is driven into a NDC state, the nonlinearities can be further enhanced by the onset of high-field domains and the related propagation phenomena in a similar way as the electric field domains in bulk semiconductors. In other words, the ultrafast creation and annihilation of electric domains during the period of an oscillating field contributes to HHG processes in SSLs. This dynamic process depends on plasma effects induced by the space-charge instabilities and the dielectric relaxation time processes which dictate the exact conditions for the NDC state^25,26^. The THz response from Bloch oscillations in a miniband SSL, under the influence of a THz field, might also deviate due to strong excitonic effects^27,28^. All these extra processes can be exploited to enhance the nonlinearities driving HHG in SSLs, but in this paper we focus on assuming that uniform static and oscillating input fields modulate the Bloch oscillations, which has very successfully predicted both off and even harmonics in unbiased SSLs^7,8^. Furthermore, the experimental data in Fig. 2c allows us to identify a critical voltage for the whole structure of 18 period superlattices, $V_{c,T} = { }$ 429.84 mV, which corresponds in Eqs. (1) – (4) to $V_{c} =$ 23.88 mV (critical voltage per period) characterizing the NDC range. Using the characteristic vacuum impedance $Z_{0} =$ 377 Ohms and the connection between power and voltage, we obtain a critical power $W_{c} = V_{c,T}^{2} /Z_{0} =$ 0.49 mW, fully consistent with the HHG high power regions in both experiments and theoretical predictions shown in Figs. 1 and 3. This further confirms the validity of our model. Under illumination, the voltages cannot go above 100 mV without significant damage to the samples and that is why we do not see the development of the “petals” above 200 mV illustrated in Fig. 1. As a matter of fact, we have modulated Bloch Oscillations and for input frequencies with energy $h\nu$ much larger than $\Gamma^{ + } \sim { }\Gamma^{ - }$ the maxima correspond to resonances between Bragg reflections and the harmonic frequencies: $\omega_{B} = 2\pi l\nu$ in an idealized system. We have tested the simulations for high frequencies and indeed we see these resonances plus an extra resonance corresponding to the negative difference resistance energy, i.e. $\sim { }\Gamma^{ + } \sim { }\Gamma^{ - }$. For low frequencies the maxima appear at intermediate values between $2\pi l\nu$ and multiples of $\sim { }\Gamma^{ + } \sim { }\Gamma^{ - }$. This renormalization energy type of effect will be the subject of future investigations with other samples and resonators capable of sustaining higher frequency input and output.

Our model is further confirmed by Fig. 4, which zooms in low voltages. The calculations show that asymmetric flow is required to explain the even order HHG at zero bias found experimentally, see Fig. 3a,b. Note that our waveguides cut the second harmonic emission, to avoid saturation at the detector level, since our experimental focus is on higher harmonics.

At this point, we should note that It is known in the literature that the interfaces of GaAs over AlAs do not have the same quality as those of AlAs over GaAs and we successfully modelled the difference using an interface roughness model, explaining even harmonic emission without bias^6,7^. A clear picture of the difference between interfaces can be seen, e.g. in Ref.^29^. Figure 2c shows that there are some deviations between our ideal model and the actual I–V of the superlattice under study. Notably, around zero bias, the experimental I–V is a lot more linear than the Esaki–Tsu-like shape predicted both by NEGF calculations and by the Boltzmann Equation approach^7,8^. Note this is a common feature in the literature and the same effect has been seen by different authors and samples^7,30^. Nevertheless, the averaged input parameters used reproduce the main harmonic generation experimental findings as given in Fig. 3. The averaged parameters were used in our Ansatz solution of asymmetric I–V flow^7,8^.

In conclusion, we have experimentally and theoretically investigated the important interplay of asymmetric current flow, applied static voltages and GHz input power on GHz-THz nonlinearities in semiconductor superlattices. The experimental results are a clear-cut demonstration of our theoretical predictions that the usual susceptibility concept cannot be used. Quite on the contrary, we have demonstrated giant control and enhancement of emitted harmonic power. The harmonics reach maxima or disappear completely with relatively small changes of voltages and GHz input power. Superlattices are used both as nonlinear sources and in the fast heterodyne detection scheme, with the whole set operating real time at room temperature and with low powers and voltages compatible with compact devices. Our combined theory and experimental approach opens the door to investigate a plethora of nonlinear effects under controlled conditions in the GHz–THz range. The even harmonics enhancement per voltage control, makes the efficiency of superlattice multipliers much larger than the recently predicted values for unbiased structures. This effort will also lead to further understanding and development of practical GHz-THz sources and detectors operating in a range where Quantum Cascade Lasers and other optical sources will most likely never operate without cryocooling.

## Methods

### Experimental setup

The superlattices used in the experiments have a structure similar to those used in the experiments in Refs.^7,8^, namely: 18 periods of 6.23 nm each with 18 monolayers GaAs and four monolayers AlAs. The samples are homogeneously doped with silicon with a density around 2 × 10^18^ cm^−3^.

### Current–voltage

The experimental I–V, see blue symbols in Fig. 2c, is measured with an oscilloscope in XY mode (without time sweep). Voltage from a signal generator, (in either sawtooth or sine form), with a frequency of about 1 kHz is applied to the SSL and load resistor which are connected in series. The X-channel oscilloscope input was fed with voltage from SSL diode. The Y-channel was fed with voltage from resistor with a high bandwidth voltage follower. This measurement allows to check the input parameters for the simulations of harmonic power. For teams interested in nonlinear optics, but without access to NEGF or Boltzmann equation or NEGF solvers^5,6^, the peak currents and voltages can be extracted from the static I–V and used as input for our analytical expressions.

### High harmonic generation and detection

In our innovative scheme, SSLs are used both as emitter of the nonlinear output to be analyzed and as the main element in detection, by means of a fast heterodyne detection at room temperature. Applied voltage to the SSLM is provided by a bias unit based on a battery and resistors. This avoids interference from supply lines. A voltmeter is attached in parallel with the bias unit for voltage control. The power sources for the experiments are two computerized Backward Wave Oscillator (BWO) based synthesizers with frequency range 118–170 GHz, delivering a few decades of power (from 20 up to 60 mW). Input power to the SSL under study is delivered through a waveguide (WG) output flange, followed by an adjustable WG attenuator to control the input power. All WGs used are of rectangular WR-6 type (1.651 × 0.8255 mm). The WG chamber housing the SSL has also a DC port (SMA connector) to deliver the controlling bias required by the study. The multiplier and detector chambers have horn antennas for input and output. Both antennas block radiation below 400 GHz to prevent the 1st (pump power) and 2nd harmonic radiation, which would be too intense and mask detection of the higher order harmonics, which are the subject of investigation in this work. The multiplier and mixer units are arranged horn-to-horn at a distance that optimizes the detection. For the waveguides used, we have about 5 mm distance between antennas. The second BWO based synthesizer was used as local oscillator (LO) for pumping the frequency mixer, together with its own attenuator to set the mixer in optimal regime. The output SMA port of the SSL mixer is connected to an RF amplifier chain, which delivers 50 dB amplification, with a 2 dB noise factor and 20 MHz to 2 GHz amplification frequency range. The resulting amplified signal at the intermediate frequency is selected by a spectrum analyzer.

In contrast with measurements using Fourier Transform (FTIR) spectrometers, our method is real time and performed at room temperature. Note that FTIR spectroscopy needs liquid He cooled bolometers for GHz-THz signal detection^7,8^. Further development of our method is a clear technological advantage, with SSLs from the same wafer used to make both sources and detectors in advanced sensors.

Both synthesizers have narrow spectral linewidth (about 1 kHz), thus allowing detection of up to the 6^th^ harmonic from the SSLM. Our spectrum analyzer has a few Hz of resolution bandwidth (RBW), but reducing the RBW of our spectrum analyzer below 10 kHz does not improve signal to noise ratios, since the emitted signals at 4th and 6th harmonics have about 10 kHz linewidth. The experimental setup is summarized in Fig. 2.

### Heterodyne beat signal

In the heterodyne detection scheme considered here, the semiconductor superlattice (SSL) nonlinear mixer medium is exposed to a total field $E\left( t \right),{ }$ comprised of a static bias, a local oscillator LO with frequency ${\Omega }_{1}$ and the signal from the SSLM under study with frequency $\Omega _{2}$. The LO is an independent second BWO with a Phase Locked Loop (PLL). The SSLM signal contains all generated harmonics and the input signal. The WGs suppress the fundamental frequency and second harmonic emerging from the SSLM. We also include an arbitrary dephasing $\phi_{2}$ between the harmonic under study and the LO. The PLL circuit locks this phase to a constant value.

5 $$F\left( t \right) = F_{dc} + F_{1} cos\left( {{\Omega }_{1} t} \right) + F_{2} cos\left( {{\Omega }_{2} t + \phi_{2} } \right).$$

The nth harmonic from the SSLM is mixed with the slightly detuned nth harmonic from the LO and a number of beatings develop at intermediate frequencies (IFs) $\Omega _{i} = \left| {k{\Omega }_{1} + p{\Omega }_{2} } \right|$, where k and p can be either positive or negative integers. We have chosen an IF signal frequency region free of interference from microwave radio communications (GSM, 4G etc.). The matching conditions for detecting the nth harmonic are achieved by selecting the following terms $p = 1,{ }k = - n{ };p = - 1,{ }k = n.$

The current at the desired intermediated (IF) frequency ${\Omega }_{i} \equiv {\Omega }_{2} = n{\Omega }_{BWO} - n{\Omega }_{1}$ reads.

6 $$I_{{{\Omega }_{i} }} = I_{{{\Omega }_{i} }}^{c} \cos \left( {{\Omega }_{i} t + \phi_{2} } \right) + I_{{{\Omega }_{i} }}^{s} \sin \left( {{\Omega }_{i} t + \phi_{2} } \right).$$

If the harmonic field is sufficiently small, $\alpha_{2} = \frac{{eE_{2} {\text{d}}}}{{\hbar {\Omega }_{2} }} \ll 1$, the exact solution for $I_{{{\Omega }_{i} }}$ can be expanded and it can be shown that it depends linearly on the amplitude of the harmonic field. The RMS valued detected by the spectrum analyzer can then be written as.

7 $$I^{RMS} = \sqrt {I_{{{\Omega }_{i} }}^{2} } = {\mathcal{C}}E_{2} ,dbn_{E} = 10\log \left( {\frac{{E_{2} }}{{E_{max} }}} \right) = 10\log \left( {\frac{{I^{RMS} }}{{I_{max}^{RMS} }}} \right).$$

### Calibration-input power

We have no direct access to the value of the electric field inside the structure, $E_{ac}$ and have thus calibrated the value of electric field and power inside the superlattice by extending the method used in Refs.^7,8^. For this we developed a dedicated simulated annealing algorithm. This is based on a meta heuristic technique to approximate the global minimum in a large search space whereby one mimics the annealing procedure from metallurgy that finds the minimal energy of a thermodynamical system^31,32^. In contrast with Refs.^7,8^ that had only one input power value, we have here multiple input power values. For each input power, we take the set of outputs for the variable voltage and create a data set. To this data set we apply simulated annealing and find the best value of the power related parameter $\alpha$ inside the superlattice. Next we convert $\alpha$ to power in mW assuming a plane wave. The connection between input power (in mW) and $\alpha = \frac{{eE_{ac} d}}{h\nu }$ is given by $P_{in} \left( {mW} \right) = 2.941{ } \times { }10^{ - 9} { }\nu^{2} \alpha^{2}$. This delivers a calibration technique connecting the field immediately outside the waveguide and the internal field $E_{ac}$. The numbers obtained are perfectly consistent with the $\alpha$ values similarly determined in Refs.^7,8^. This is as expected, since the waveguides have identical structure, so the losses from the flange of the waveguide to the interior where the superlattice is housed, should be similar.

### Output power

Furthermore, for the same waveguides, we have experimentally demonstrated that higher input frequencies require higher input powers to achieve roughly the same field inside the SSLM. From Ref.^8^, input frequencies $\nu =$ 130, 140, 150 and 160 required, respectively BWO input powers of 1.8, 4.1, 11.2 and 33.9 mW to deliver input powers inside the superlattice corresponding to $\alpha$ = 35.2, 28.3, 26.1 and 23.7.

This means that if we take 4.1 mW input at $\nu = { }$ 120 GHz, as in the experiments in this paper, we expect an alpha value larger than 28.3 within the same order of magnitude inside the superlattice and indeed our calibration yields $\alpha$ = 38.3, which is perfectly consistent, since the waveguides are indeed identical. This allows us to move a step further and calibrate the heterodyne detection method also for the output powers by comparison with the outputs measured in Ref.^7^. We adjust the output map based on the following scaling: 4th Harmonic: $P_{4H}^{exp}$ = $4.75{ } \times 10^{ - 9}$ W at an input frequency $\nu = 141$ GHz and $\alpha = 28.3$; 6^th^ Harmonic: $P_{6H}^{exp}$ = $3.85{ } \times 10^{ - 9}$ W at and input frequency $\nu = 141$ GHz and $\alpha = 28.3$. The ratios of the transmission of the waveguides for the 4th and 6th harmonics between $\nu = 120$ GHz used here and $\nu = 141$ GHz are given, respectively by $wg_{4H} \left( {120/141} \right) = 0.89{ }$ and ${ }wg_{6H} (120/141) = 0.97.$ Note further that the calculated powers are proportional to the square of the static peak current $j_{PRB}^{ + }$ = 2.7 mA and here we have $j_{0}^{ + }$ = 7.4 mA. We can then use our numerical scheme to calculate reference values ${\mathcal{P}}_{4H} \left( {141,28.3} \right)$ and ${\mathcal{P}}_{6H} \left( {141,28.3} \right)$.

Thus for a given input power at $\nu = 120$ GHz, characterized by the parameter $\alpha$, we use our numerical scheme to calculate the output powers of both harmonics, i.e. ${\mathcal{P}}_{4H} \left( {120,\alpha } \right)$ and ${\mathcal{P}}_{6H} \left( {120,\alpha } \right)$.

The calibration factors are:

8 $$\begin{aligned} & {\mathcal{F}}_{4H} = P_{4H}^{exp} { }wg_{4H} \left( {120/141} \right){ }\left( {j_{0}^{ + } /j_{PRB}^{ + } } \right)^{2} { }/{\mathcal{P}}_{4H} \left( {141,28.3} \right); \\ & {\mathcal{F}}_{6H} = P_{6H}^{exp} { }wg_{6H} \left( {120/141} \right){ }\left( {j_{0}^{ + } /j_{PRB}^{ + } } \right)^{2} { }/{\mathcal{P}}_{6H} \left( {141,28.3} \right) \\ \end{aligned}$$

The final output powers (in μW) are thus given by.

9 $$P_{4H}^{out} \left( \alpha \right) = {\mathcal{F}}_{4H} { }{\mathcal{P}}_{4H} \left( {120,\alpha } \right)\quad {\text{and}}\quad P_{6H}^{out} \left( \alpha \right) = {\mathcal{F}}_{6H} { }{\mathcal{P}}_{6H} \left( {120,\alpha } \right)$$

Regarding the experimental data, important is to find the maximum contrast between minimum and maximum. For a given input power, the detected power with the heterodyne detection scheme is given for each harmonic by ${\mathfrak{P}}_{4H}^{exp} \left( \alpha \right)$ and ${\mathfrak{P}}_{6H}^{exp} \left( \alpha \right)$.

We thus find the experimental maxima, ${\mathfrak{P}}_{4H}^{exp,max}$ and ${\mathfrak{P}}_{6H}^{exp,max}$ and use a similar scaling.

10 $$P_{4H}^{out,exp} \left( \alpha \right) = \left\{ {{\mathfrak{P}}_{4H}^{exp} \left( \alpha \right)/{ }{\mathfrak{P}}_{4H}^{exp,max} } \right\}P_{4H}^{out,max} ,P_{6H}^{out,exp} \left( \alpha \right) = \left\{ {{\mathfrak{P}}_{6H}^{exp} \left( \alpha \right)/{ }{\mathfrak{P}}_{6H}^{exp,max} } \right\}P_{6H}^{out,max} .$$

This clearly put numbers to further highlight orders of magnitude control and enhancement delivered by a combination of input power and voltage control. We obtain μW peak powers, in contrast with the nW peak powers detected in Refs.^6,7^, with zero voltage and all even harmonic power output explained uniquely by asymmetric current flow.

## Data Availability

All data that support the findings of this study are present in the paper and/or the Supplementary Materials. Additional data related to this paper may be requested from the authors upon reasonable request.

## Code availability

The code that was used to simulate the findings of this study is available from the corresponding authors upon reasonable request.
